# Nationwide trends in chemotherapy use and survival of elderly patients with metastatic pancreatic cancer

**DOI:** 10.1002/cam4.1240

**Published:** 2017-10-16

**Authors:** Lydia G. M. van der Geest, Nadia Haj Mohammad, Marc G. H. Besselink, Valery E. P. P. Lemmens, Johanneke E. A. Portielje, Hanneke W. M. van Laarhoven, J. (Hanneke) W. Wilmink, the Dutch Pancreatic Cancer Group

**Affiliations:** ^1^ Department of Research Netherlands Comprehensive Cancer Organisation (IKNL) Utrecht The Netherlands; ^2^ Department of Medical Oncology University Medical Center Utrecht Utrecht The Netherlands; ^3^ Department of Surgery Academic Medical Center Amsterdam The Netherlands; ^4^ Department of Public Health Erasmus Medical Center Rotterdam The Netherlands; ^5^ Department of Internal Medicine and Medical Oncology Haga Hospital The Hague The Netherlands; ^6^ Foundation of Geriatric Oncology Netherlands (GeriOnNe) Eindhoven The Netherlands; ^7^ Department of Medical Oncology Academic Medical Center Amsterdam The Netherlands

**Keywords:** Chemotherapy, distant metastasis, elderly, pancreatic adenocarcinoma, survival

## Abstract

Despite an aging population and underrepresentation of elderly patients in clinical trials, studies on elderly patients with metastatic pancreatic cancer are scarce. This study investigated the use of chemotherapy and survival in elderly patients with metastatic pancreatic cancer. From the Netherlands Cancer Registry, all 9407 patients diagnosed with primary metastatic pancreatic adenocarcinoma in 2005–2013 were selected to investigate chemotherapy use and overall survival (OS), using Kaplan–Meier and Cox proportional hazard regression analyses. Over time, chemotherapy use increased in all age groups (<70 years: from 26 to 43%, 70–74 years: 14 to 25%, 75–79 years: 5 to 13%, all *P* < 0.001, and ≥80 years: 2 to 3% *P* = 0.56). Median age of 2,180 patients who received chemotherapy was 63 years (range 21–86 years, 1.6% was ≥80 years). In chemotherapy‐treated patients, with rising age (<70, 70–74, 75–79, ≥80 years), microscopic tumor verification occurred less frequently (91‐88‐87‐77%, respectively, *P* = 0.009) and OS diminished (median 25‐26‐19‐16 weeks, *P* = 0.003). After adjustment for confounding factors, worse survival of treated patients ≥75 years persisted. Despite limited chemotherapy use in elderly age, suggestive of strong selection, elderly patients (≥75 years) who received chemotherapy for metastatic pancreatic cancer exhibited a worse survival compared to younger patients receiving chemotherapy.

## Introduction

Pancreatic cancer is one of the most dismal types of cancer, with a 5‐year survival rate of only 5–7% 1, 2. These low survival rates reflect an advanced stage at diagnosis in the vast majority of patients: at least half of patients already have metastatic disease at time of diagnosis 3, 4. Median survival of unselected patients with metastatic disease is only 2–3 months 3, 4, 5.

Pancreatic cancer is predominantly a disease of the elderly 2, 6, at least half of all patients are over 70 years of age and more than one‐fifth is older than 80 years 6, 7. Unfortunately, elderly patients are underrepresented in clinical trials. For example, the phase III study which showed that FOLFIRINOX (oxaliplatin, irinotecan, fluorouracil, and leucovorin) significantly improved survival compared with gemcitabine monotherapy (median survival 11.1 vs. 6.8 months, respectively) excluded patients over 75 years of age 8. The phase III study on the combination of gemcitabine and nab‐paclitaxel included patients until 88 years of age (median survival 8.5 months vs. 6.7 months in patients with gemcitabine‐alone), but the median age of 63 years suggests that few patients were older than 75 years 9.

Population‐based studies have shown that in the past decades the administration of palliative chemotherapy steeply increased in patients with metastatic pancreatic cancer 3, 10. Whether the increased use of chemotherapy also applies to elderly patients, is unknown. Furthermore, some specialized institutions have reported acceptable safety and efficacy of chemotherapy in selected elderly patients with metastatic pancreatic cancer, with survival comparable with younger patients 11, 12, 13. However, in these reports a direct comparison with younger patients (<75 years) was not performed 11, 12 or a single age cut‐off (<70, ≥70 years) was used 13 which may mask variation within the older age group. To the best of our knowledge, no population‐based studies have been published which compare survival after chemotherapy according to age.

Therefore, the purpose of this nationwide study is to examine the use of chemotherapy and its impact on overall survival in elderly patients with metastatic pancreatic cancer, using multiple age groups.

## Methods

### Netherlands cancer registry

In the Netherlands, a country with approximately 16.8 million inhabitants, all newly diagnosed malignancies are recorded in the nationwide Netherlands Cancer Registry (NCR). Besides notification by the automated pathological archive (PALGA), the National Registry of Hospital Discharge Diagnoses is used. Subsequently, trained registrars collect information on patient, tumor and primary treatment from the medical records in all Dutch hospitals. The International Classification of Diseases for Oncology (ICD‐O‐3) is used for coding of morphology and tumor locations 14. Histologically confirmed malignancies are staged according to the Tumor‐Node‐Metastasis (TNM) staging classification 15. In patients without microscopically verified diagnosis a summary stage is recorded (Extent of Disease, EoD). Data quality is high and completeness is estimated to be at least 95%. Follow‐up for all patients is obtained by routinely linking the NCR to the Municipal Personal Records Database (BRP). The BRP contains information on the vital status of all Dutch inhabitants (dead or alive, date of death or emigration). The NCR Review Board approved the study.

### Patients

For this study, from the NCR all patients were selected who were diagnosed with primary invasive pancreatic (ductal) adenocarcinoma in the period 2005–2013 (ICD‐O‐3 C25, morphology codes 8010, 8012, 8020, 8140, 8141, 8260, 8310, 8440, 8470, 8480, 8481, 8490, 8500, 8560, or a nonmicroscopic verified invasive neoplasm of the pancreas suspected for adenocarcinoma). Patients diagnosed at autopsy, younger than 18 years or residing abroad were excluded. TNM and EoD staging information were combined to select patients with metastatic disease at diagnosis (53% of patients).

To investigate a possible age gradient or age cut‐off point, patients were divided into four age groups: <70 years, 70–74 years, 75–79 years and ≥80 years of age. Due to the nature of the NCR, information on prior primary malignancies was available in all patients. Additionally, a slightly modified version of the Charlson classification was recorded region‐wide within 1–2 out of nine cancer regions (18% of all patients). Serious comorbid conditions included chronic obstructive pulmonary diseases, cardiovascular diseases, cerebrovascular diseases, digestive tract diseases, diabetes mellitus and other serious diseases. The number of comorbidities were categorized in three groups (0, 1, ≥2). Furthermore, data on socioeconomic status (SES) were used 16. SES was based on reference data from The Netherlands Institute for Social Research. Scores on social deprivation were derived from income, education and occupation per 4‐digit postal code, and were broken into three SES‐categories (high: 1st–3rd, intermediate: 4th–7th, low: 8th–10th deciles). Registered treatment after diagnosis included the cancer treatment modalities as mentioned in the treatment plan and provided to the patient (i.c. resection, radiotherapy, chemotherapy). Time intervals between date of diagnosis and date of initiating chemotherapy were calculated to explore possible delay.

Survival time was calculated from the date of diagnosis to the date of death or 1 January 2015, whichever came first. To reduce the influence of survivor treatment selection bias in analysis of survival of patients with versus without chemotherapy 17, only patients were selected who survived at least 30–days after diagnosis (conditional survival). In addition to information about delay of starting chemotherapy, survival time from the starting date of chemotherapy was calculated.

### Statistical analysis

In each age group of patients with metastatic pancreatic cancer, Chi square tests for trend were performed to assess the administration of chemotherapy in consecutive 3‐year periods (2005–2007, 2008–2010, 2011–2013). A two‐sided *P* < 0.05 was considered statistically significant. In patients receiving palliative chemotherapy, Chi‐square tests were also used to compare patient, tumor and treatment characteristics between age groups. To compare time intervals between groups of patients, nonparametric Kruskal–Wallis tests were used. Univariable and multivariable logistic regression analyses were performed to investigate the association of patient and tumor characteristics with the administration of chemotherapy. Kaplan–Meier analyses with log rank tests were used (1) to evaluate overall survival of all patients with metastatic disease and (2) to compare overall and conditional survival of chemotherapy‐treated and untreated patients within the different age groups. In patients receiving chemotherapy, univariable and multivariable Cox proportional hazard regression analyses were performed to evaluate predictors for a worse survival, using survival time calculated from (1) date of diagnosis and from (2) starting chemotherapy. In multivariable models, a backward stepwise elimination procedure was used with a *P* > 0.10 in likelihood ratio tests for removal of variables. Missing values were included as separate categories or dummy variables. In sensitivity analyses using region‐wide data only, the additional influence of the number and type of comorbid conditions was investigated (in addition to the predictors derived from the multivariable models in the total population). All analyses were performed using STATA/SE (version 13.0; STATA Corp., College Station, TX).

## Results

### Patients with metastatic pancreatic cancer

**Figure 1:**
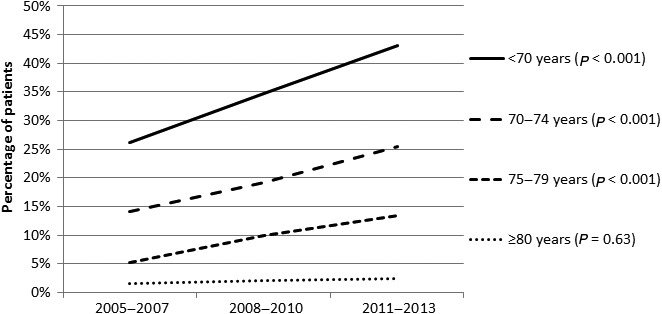
Administration of chemotherapy to patients diagnosed with metastatic pancreatic cancer in consecutive time periods in the Netherlands, by age group.

Of 9,407 patients diagnosed with metastatic pancreatic cancer in the period 2005–2013, 32% was 75 years or older. Twenty‐three per cent of all patients received palliative chemotherapy. Over time, the administration of palliative chemotherapy more than doubled from 13% in 2005 to 30% in 2013 (*P* < 0.001). Although treatment with chemotherapy was far less common in elderly patients, an increased use of chemotherapy was found in all age groups (Fig. 1). In consecutive 3‐year periods, from 26% to 43% of patients under age 70 years received chemotherapy, from 14% to 25% of patients aged 70–74 years and from 5% to 13% of patients aged 75–79 years (all *P* < 0.001). Over age 80 years very few patients were treated with chemotherapy and the very small increase of 2–3% was not statistically significant (*P* = 0.56). Besides elderly patients (≥70 years) and patients diagnosed in earlier years, also patients living in low SES neighborhoods, without tumor verification, with tumors located in the pancreatic head and patients with multiple metastatic sites independently had a lower probability of receiving chemotherapy (Table 1), In addition, the accumulation of comorbid conditions showed a stronger association with not receiving chemotherapy than specific comorbid conditions.

**Table 1:** Characteristics of patients with primary pancreatic adenocarcinoma and synchronous distant metastases in the period 2005‐2013 in the Netherlands, by administration of chemotherapy (CT) and logistic regression analyses predicting administration of chemotherapy

	Patients	CT	Univariable analysis		Multivariable analysis	
	*N* = 9407 (%)	%	OR (95% CI)	*P* ‐value	OR (95% CI)	*P*‐value
Age				<0.001		<0.001
<70 years	4729 (50)	35	1.00		1.00	
70–74 years	1623 (17)	20	0.46 (0.41–0.53)		0.49 (0.43–0.57)	
75–79 years	1437 (15)	9.9	0.20 (0.17–0.24)		0.23 (0.19–0.28)	
≥80 years	1618 (17)	2.2	0.04 (0.03–0.06)		0.06 (0.04–0.09)	
Year of diagnosis	9407 (100)	23	1.12 (1.10–1.15)	<0.001	1.12 (1.10–1.15)	<0.001
Gender						
Male	4852 (52)	25	1.00	0.001		
Female	4555 (48)	22	0.85 (0.77–0.93)			
History of cancer						
No	8104 (86)	24	1.00	<0.001	1.00	0.07
Yes	1303 (14)	18	0.70 (0.60–0.81)		0.86 (0.73–1.01)	
SES						
High	2,839 (30)	26	1.00	<0.001	1.00	0.006
Intermediate	3,736 (40)	23	0.89 (0.80–1.00)		0.95 (0.84–1.07)	
Low	2,832 (30)	21	0.76 (0.67–0.86)		0.81 (0.71–0.93)	
Tumor verification				<0.001		<0.001
Verified	6,486 (69)	30	1.00		1.00	
No verification	2,921 (31)	7	0.18 (0.15–0.21)		0.29 (0.25–0.34)	
Primary tumor				<0.001		
Head of pancreas	4567 (49)	21	1.00		1.00	
Body or tail	3254 (35)	27	1.42 (1.28–1.57)		1.33 (1.19–1.49)	<0.001
Overlapping/NOS	1586 (17)	20	0.95 (0.83–1.10)		0.98 (0.84–1.14)	
Metastatic site						
1	6283 (67)	24	1.00	<0.001	1.00	<0.001
≥2	2808 (30)	23	0.95 (0.86–1.06)		0.79 (0.70–0.89)	
Unknown	316 (3.4)	12	0.44 (0.31–0.62)		0.62 (0.43–0.90)	
*Sensitivity analysis* a						
Comorbid c.	(*n* = 1697)			<0.001	b	0.06
0	420 (25)	36	1.00		1.00	
1	466 (27)	26	0.63 (0.47–0.83)		0.78 (0.57–1.07)	
≥2	590 (35)	19	0.40 (0.30–0.54)		0.67 (0.48–0.94)	
Unknown	221 (13)	16	0.34 (0.23–0.52)		*‐*	
Comorbid c.	(% yes)		(yes vs. no)		b	
Diabetes	394 (27)	23	0.82 (0.63–1.08)	0.16		
Cardiac	353 (24)	15	0.43 (0.32–0.60)	<0.001		
Vascular	271 (18)	17	0.51 (0.36–0.72)	<0.001	0.69 (0.47–1.04)	0.07
Pulmonary	170 (12)	18	0.58 (0.38–0.87)	0.009		
Hypertension	450 (31)	23	0.79 (0.61–1.02)	0.07		
Digestive tract	151 (10)	28	1.11 (0.76–1.61)	0.60		

CT, chemotherapy; Comorbid c., Comorbid conditions; SES, socioeconomic status; NOS, not otherwise specified; OR, odds ratio; CI, Confidence Interval.

a Region‐wide data only *n* = 1697 (18% of all patients). Multivariable model adjusted for variables included in model using nationwide data (age, year of diagnosis, history of cancer, SES, tumor verification, primary tumor and number of metastatic sites).

b Excluding *n* = 221 patients with unknown comorbid conditions because of collinearity.

**Figure 2:**
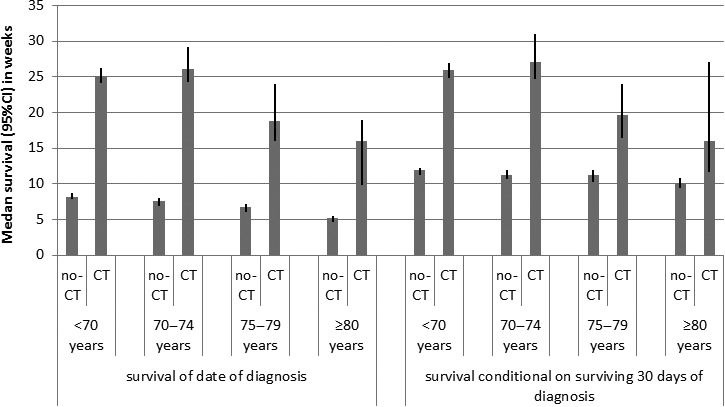
Median overall survival and conditional overall survival with 95% confidence interval of patients who received palliative chemotherapy (CT) for metastatic pancreatic cancer compared with untreated patients (no‐CT), by age group.

Median overall survival (OS) of patients with metastatic pancreatic cancer was 9.5 weeks (with rising age of patients [<70, 70–74, 75–79, ≥80 years]: 13‐10‐8‐5 weeks, respectively, *P* < 0.001), and OS was 7 weeks in untreated patients versus 25 weeks (5.7 months) in patients who received chemotherapy (*P* < 0.001). As many as 26% of all patients died within 30 days after diagnosis (with rising age: 19‐26‐32‐43%, *P* < 0.001). In patients who survived 30 days, Chemotherapy‐treated patients under 75 years survived longer compared to untreated patients (conditional survival [CS] <70 years: median 26 vs. 12 weeks, 70–74 years: 27 vs. 11 weeks, both *P* < 0.001), but the survival difference was smaller in patients over 75 years of age (75–79 years: median 20 vs. 11 weeks, *P* < 0.001, ≥80 years: 16 vs. 10 weeks, *P* = 0.02, Fig. 2).

### Patients receiving systemic chemotherapy

Median age of 2,180 patients who received palliative chemotherapy for metastatic pancreatic cancer was 63 years (range, 21–86 years) and increased from 62 years in 2005–2007 to 64 years in 2011–2013. Eight per cent of treated patients were 75 years or older and only few patients were over 80 years of age (*n* = 35, 1.6%). With rising age (<70, 70–74, 75–79, ≥80 years), the prevalence of a prior cancer diagnosis and the number of comorbid conditions increased (both *P* < 0.001), particularly cardiac and vascular diseases (*P* < 0.001 and *P* = 0.001, respectively, Table 2). Furthermore, older patients less often had microscopic verification of the current cancer (91%, 88%, 87%, 77%, respectively, *P* = 0.009), although they all received chemotherapy.

**Table 2:** Characteristics of patients who received palliative chemotherapy (CT) for metastatic pancreatic cancer in the period 2005–2013 in the Netherlands, by age group

	All patients	<70 years	70–74 years	75–79 years	≥80 years	Chi^2^
	*N* = 2180%	*N* = 1674%	*N* = 329%	*N* = 142%	*N* = 35%	*P*–value
*Patient*						
Gender						
Male	1194 (55)	56	51	54	57	0.49
Female	986 (45)	44	49	46	43	
Socioeconomic status (SES)						
High	724 (33)	33	34	29	40	
Intermediate	874 (40)	40	38	42	46	0.56
Low	582 (27)	27	28	29	14	
History of cancer						
No	1944 (89)	91	85	77	77	<0.001
Yes	236 (11)	8.7	15	23	23	
Comorbid c.^a^	(n = 420)	(n = 325)	(n = 64)	(n = 27)	(n = 4)	<0.001
0	152 (36)	42	17	22	0	
1	122 (29)	30	25	26	50	
2+	110 (26)	20	52	37	25	
Unknown	36 (8.6)	8.3	6.3	15	25	
Comorbid c. (%yes)^a^	(*n* = 384)^b^	(*n* = 298)^b^	(*n* = 60)^b^	(*n* = 23)^b^	(*n* = 3)^b^	
Diabetes	92 (24)	21	35	30	0	0.09
Cardiac disease	54 (14)	9.7	28	26	67	<0.001
Vascular disease	45 (12)	8.4	25	17	33	0.001
Pulmonary disease	30 (7.8)	7.1	13	4.4	0	0.33
Hypertension	103 (27)	24	40	26	33	0.09
Digestive tract disease	42 (11)	9.7	10	26	33	0.06
*Tumor*						
Tumor verification						0.009
Verified	1968 (90)	91	88	87	77	
No verification	212 (9.7)	8.8	12	13	23	
Primary tumor						0.27
Head of pancreas	964 (44)	44	48	44	34	
Body or tail	894 (41)	42	36	39	57	
Other or overlapping	322 (15)	15	16	17	8.6	
Number of metastatic sites						
1	1497 (69)	69	69	63	69	0.51
≥2	645 (30)	29	28	37	31	
Unknown	38 (1.7)	1.8	2.1	0.7	0	
Time interval to CT						
Median [p25–p75] in days^c^	25 [15–42]	26 [15–43]	26 [16–48]	21 [13–33]	18 [13–34]	0.007
≥6 weeks^c^	443 (26)	27	29	17	12	0.03
Mortality of starting CT						
30–day mortality^c^	141 (8.4)	8.0	8.5	10.0	20.0	0.17
90–day mortality^c^	541 (32)	31	31	44	48	0.02

Comorbid c., Comorbid conditions; SES, socioeconomic status; NOS, not otherwise specified; N, number of patients.

a Region–wide data only n = 420 (18% of all patients).

b excluding patients with unknown comorbid conditions.

c If date of initiating chemotherapy is available *n* = 1676 (77% of all patients).

The date of initiation of chemotherapy was available in 77% of patients and characteristics of these patients did not differ from the total group of patients (data not shown). Median time‐to‐chemotherapy was 25 days ([p25‐p75] 15–43 days) and elderly patients started chemotherapy sooner after diagnosis (Table 2), as well as patients with nonhead tumors (head: median 32 days, nonhead: 21, *P* < 0.001) and patients with at least two metastatic sites (1: median 27 days, ≥2: 22, *P* < 0.001).

With rising age (<70, 70–74, 75–79, ≥80 years), median OS of treated patients decreased: 25, 26, 19, and 16 weeks, respectively, (*P* = 0.003). In univariable survival analysis of patients who received chemotherapy, a higher probability of worse OS was found in patients over 75 years of age, patients treated in earlier years of our study period, without microscopic tumor verification, with nonhead cancer, and in patients with multiple metastatic sites (Table 3). In the multivariable Cox proportional hazard model, all these characteristics were independently associated with a poor OS. Compared with chemotherapy‐treated patients younger than 70 years of age, patients over 75 years of age who received chemotherapy showed a worse OS (Hazard Ratio [HR] (75–79 vs. <70) = 1.21, 95% CI 1.02–1.44; HR (≥80 vs. <70) = 1.48, 95% CI: 1.06–2.07), but the intermediate age group did not (HR [70–74 vs. <70]=0.92, 95% CI: 0.81–1.03, *P* = 0.16). In sensitivity analysis, the number and type of comorbid conditions of treated patients seemed not significantly associated with a poor OS (borderline: pulmonary diseases: adjusted HR = 1.38, 95% CI: 0.94–2.01, *P* = 0.10).

**Table 3:** Crude median overall survival and univariable and multivariable Cox proportional hazards regression analyses predicting survival of patients who received palliative chemotherapy for metastatic pancreatic cancer in the period 2005–2013 in the Netherlands

Characteristics	MS months	Univariable analysis		Multivarivable analysis	
		HR (95% CI)	*P* ‐value	HR (95% CI)	*P* ‐value
Age			0.003		0.008
<70 years	5.8	Ref		Ref	
70–74 years	6.0	0.93 (0.83–1.05)		0.92 (0.81–1.03)	
75–79 years	4.3	1.25 (1.05–1.48)		1.21 (1.02–1.44)	
≥80 years	3.7	1.58 (1.13–2.21)		1.48 (1.06–2.07)	
Year of diagnosis	5.7	0.98 (0.96–1.00)	0.05	0.98 (0.96–1.00)	0.03
Gender			0.30		
Male	5.5	Ref			
Female	6.2	0.96 (0.88–1.04)			
History of cancer			0.85		
No	5.7	Ref			
Yes	6.0	0.99 (0.86–1.13)			
SES					
High	5.8	Ref	0.27		
Medium	5.5	1.05 (0.95–1.16)			
Low	6.0	1.09 (0.98–1.22)			
Tumor verification			0.02		0.007
Verification	5.8	Ref		Ref	
No verification	4.9	1.19 (1.03–1.37)		1.22 (1.05–1.41)	
Primary tumor			<0.001		0.002
Head	6.2	Ref		Ref	
Body or tail	5.4	1.21 (1.11–1.33)		1.17 (1.07–1.29)	
Overlapping/NOS	5.7	1.17 (1.03–1.33)		1.17 (1.03–1.33)	
Metastatic sites			<0.001		<0.001
1	6.2	Ref		Ref	
≥2	5.0	1.38 (1.25–1.51)		1.36 (1.23–1.49)	
Unknown	5.6	1.13 (0.82–1.56)		1.07 (0.76–1.49)	
*Sensitivity analysis* a					
Comorbid c.			0.06	b	
0	5.8	Ref			
1	6.0	0.97 (0.76–1.23)			
≥2	5.4	1.18 (0.96–1.51)			
Unknown	6.3	0.71 (0.49–1.03)			
Comorbid c.	(if yes)	(yes vs. no)		b	
Diabetes	5.8	1.10 (0.87–1.40)	0.42		
Cardiac	5.2	0.97 (0.72–1.30)	0.84		
Vascular	4.8	1.02 (0.74–1.40)	0.90		
Pulmonary	5.4	1.40 (0.96–2.03)	0.10	1.38 (0.94–2.01)	0.10
Hypertension	5.8	1.16 (0.92–1.46)	0.22		
Digestive tract	7.1	0.84 (0.61–1.16)	0.29		

Survival calculated from date of diagnosis (100% of patients).

MS, median survival; Comorbid c., Comorbid conditions; SES, socioeconomic status; NOS, not otherwise specified; HR, hazard ratio; CI, Confidence Interval.

a Region–wide data only *n* = 420 (18% of all patients). Multivariable model adjusted for variables included in model using nationwide data (age, year of diagnosis, tumor verification, primary tumor, and number of metastatic sites).

b Excluding *n* = 36 patients with unknown comorbid conditions because of collinearity.

**Figure 3:**
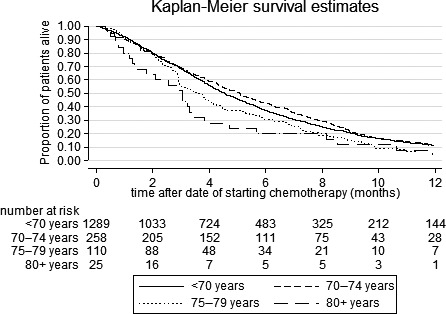
Crude overall survival of patients who received palliative chemotherapy for metastatic pancreatic cancer, by age group. Survival calculated from date of starting chemotherapy (77% of patients). Log rank test *P* = 0.006.

Using survival time calculated from the starting date of chemotherapy, median OS of treated patients was 20 weeks. With rising age (<70, 70–74, 75–79, ≥80 years), median survival was 20, 22, 16 and 13 weeks, respectively, (*P* = 0.006, Fig. 3). No survival difference was found according to tumor location (univariable HR [body/tail vs head]=1.07, 95% CI: 0.96–1.19, *P* = 0.43), but other above‐mentioned prognostic characteristics were independently associated with a worse OS (age: HR [70–74 vs <70] = 0.93, 95% CI: 0.81–1.07, HR [75–79 vs <70] = 1.24, 95% CI: 1.02–1.51, HR [≥80 vs. <70] = 1.68, 95% CI 1.13–2.50).

## Discussion

To our knowledge, this is the first nationwide study of patients with metastatic pancreatic cancer that investigated chemotherapy use and survival in multiple elderly age groups. The administration of palliative systemic therapy doubled between 2005 and 2013 in all age groups. Compared with younger patients receiving chemotherapy, treated patients over 75 years of age less often underwent microscopic tumor verification of cancer and showed a worse overall survival.

In consistent with previous population‐based reports, overall survival of patients with primary metastatic pancreatic cancer in our study was only 2–3 months 4, 5, 18. Our nationwide study also confirmed a recent regional report from the Netherlands that the administration of palliative chemotherapy has increased rapidly in the past decade 3. Chemotherapy prescription in the Netherlands, however, (overall 23%, patients surviving 30 days 31%), seemed relatively low compared with population‐based studies from the USA and France (42–54%) 10, 19, 20, 21. Although no information on the type of chemotherapy was available in our study, it is plausible that gemcitabine‐based therapies were prescribed to the vast majority of patients in the selected time period 20, 21, 22. Treatment preference for gemcitabine was mainly based on its favorable clinical benefit response (pain, performance status, weight) and toxicity profile compared to 5‐fluorouracil (5‐FU) 23, 24. Only recently, the studies by Conroy et al. on FOLFIRINOX (oxaliplatin, irinotecan, fluorouracil, and leucovorin) 8 and Von Hoff et al. on the combination of gemcitabine and nab‐paclitaxel (MPACT‐trial) 9 opened new treatment perspectives 25, 26, 27. However, despite a good performance status of included patients, prolonged survival in these studies went along with an increased risk of side effects. Possibly, modified FOLFIRINOX or gemcitabine with nab‐paclitaxel treatment may be beneficial to older patients or patients with a less favourable performance status 28.

Similar to other reports 3, 20, 21, chemotherapy use in the current study was far less likely in elderly patients with metastatic pancreatic cancer. Although the number of octogenarians receiving chemotherapy hardly increased, in the course of our study the age of patients who received palliative chemotherapy rose. In patients aged 70–74 years who received chemotherapy, tumor verification rate, timing of chemotherapy, early mortality and overall survival in our study were very similar to that of treated patients younger than 70 years. However, although very few and therefore highly selected elderly patients over 75 years of age were treated with palliative chemotherapy, a poor survival after chemotherapy was particularly found in this elderly age groups. Survival of treated elderly patients in our nationwide study (≥75 years: median 4.0 months) was strikingly worse than the median of 7–8 months in previous mono‐institutional cohorts of patients over 75 years of age 11, 12. Our observations are likely related to the nationwide character of our study with a less selective cohort of elderly patients. Furthermore, additional analyses of the MPACT‐study data showed that older age (defined as ≥65 years) was an independent predictor for both worse overall and progression‐free survival 29. Unlike older age, in our study comorbid conditions seemed not strongly associated with a worse overall survival of treated patients. Possibly, a loss of ‘functional reserve’ due to the process of aging may add to a worse survival of elderly patients, resulting in increased toxicity or reduced dose adherence and consequently reduced treatment efficacy and survival. Therefore, geriatric characteristics and co‐morbid features predictive of treatment intolerance should be better defined.

Overall, survival of the total group of patients who received palliative chemotherapy in our study was similar to other observational studies (median 5.7 vs. 5–6.4 months) 13, 20, 22. Although as many as 32% of patients in our study died within 90 days of starting chemotherapy, this may reflect the treatment goal directed at symptom management and the progressive character of pancreatic cancer. Generally, chemotherapy use in the last weeks of life is considered undesirable end‐of‐life care 30. Particularly in pancreatic cancer patients with their already poor prognosis, palliative chemotherapy may jeopardize quality of end‐of‐life care and yield a limited cost‐effectiveness 31. Therefore, a better selection of patients with pancreatic cancer who may benefit from available palliative chemotherapies is clearly needed.

Most previous observational studies only included patients with microscopically confirmed pancreatic cancer 5, 11, 13, 21, 22, 32. Although pathologic confirmation of pancreatic cancer prior to chemotherapy is strongly recommended 27, 33, one in ten of treated patients in our study started chemotherapy without prior microscopic tumor verification. Especially in elderly patients, microscopic verification was often omitted. Although in selected patients, a suspected mass on computer tomography (CT), elevated serum marker CA19‐9 and cancer‐specific symptoms may result in a high specificity for pancreatic cancer 34, misdiagnosis cannot be ruled out 35, 36.

Our population‐based study also revealed that especially patients with pancreatic head tumors started palliative chemotherapy several weeks after diagnosis (median, 4–5 weeks). Many patients with pancreatic head tumors undergo stent placement to solve bile duct obstruction 37. Other patients must recover from explorative surgical procedures 38, 39, 40. Stent dysfunction and surgical morbidity may have delayed or precluded chemotherapy in a number of patients with metastatic disease. Indeed, patients with pancreatic head cancer in our study less likely received palliative chemotherapy (21%, vs. 27% in patients with body or tail disease).

Important limitations in this population‐based study concern the availability of data. Firstly, completeness of pancreatic cancer diagnoses in the NCR was questioned recently 41. Although chemotherapy use in elderly patients and survival of untreated patients might slightly be overestimated, analyses of treated patients are expected to be highly accurate. Secondly, the NCR does not contain nationwide data on comorbid conditions and performance status of patients. However, patients who received palliative chemotherapy for pancreatic cancer may already have a relatively favorable performance status and available (region‐wide) comorbidity data did not show significant associations with a poor survival. Furthermore, this nationwide population‐wide study reflects real‐world treatment and survival patterns and also included patients without microscopic confirmation of cancer and patients who underwent pancreatic resection (0.7%). Excluding these patient groups did not alter our results. Thirdly, although conditional survival analysis has reduced survivor treatment bias (immortal time bias), treatment choice was not at random (treatment selection bias) 17. Therefore, the observed differences between treated and untreated patients are likely an overestimation of true survival differences. Finally, starting dates of chemotherapy were available in only three quarters of patients. However, patients were representative for the total patient population and the available data revealed important information about the treatment process. The recently initiated Dutch nationwide PAncreatic CAncer Project (PACAP), which combines data from the NCR with the Dutch Pancreatic Cancer Audit and Dutch Pancreatic Biobank, is expected to provide more detailed information on systemic treatment in patients with pancreatic cancer, such as type and amount of chemotherapy 42.

## Conclusions

Despite a limited chemotherapy use in elderly patients, suggestive of strong selection, especially patients over 75 years of age who received chemotherapy showed a poor survival. Improved definition of the geriatric characteristics and co‐morbid features predictive of treatment intolerance is necessary to optimize selection of elderly patients for palliative chemotherapy. In addition, appropriate chemotherapy regimens are required that are better tolerated by elderly patients.

## Conflict of Interest

None declared.
